# Impact of Harvest Timing and Stir-Frying on the Bioactive Compounds, Bioactivities, and Flavor of *Ziziphi Spinosae Semen*: An Integrated Analysis via GC-IMS, Electronic Sensors, and *Caenorhabditis elegans* Model

**DOI:** 10.3390/plants15101573

**Published:** 2026-05-21

**Authors:** Junguang Ning, Hanbing Zhu, Jia Tian, Li Dai, Decang Kong, Ping Liu, Jin Zhao, Lili Wang, Mengjun Liu, Zhihui Zhao

**Affiliations:** 1 College of Horticulture, Hebei Agricultural University, Baoding 071001, China; 2 College of Food Science and Technology, Hebei Agricultural University, Baoding 071001, China; 3 Research Center of Chinese Jujube, Hebei Agricultural University, Baoding 071001, China; 4 Cangzhou Natural Resources and Planning Bureau, Cangzhou 016000, China; 5 College of Life Science, Hebei Agricultural University, Baoding 071000, China; zhaojinbd@126.com

**Keywords:** *Ziziphi Spinosae Semen*, secondary metabolites, maillard reaction, *Caenorhabditis elegans*, flavor omics

## Abstract

This study investigated the comprehensive effects of harvest timing and stir-frying on *Ziziphi Spinosae Semen* (ZSS) quality using chemical profiling, *Caenorhabditis elegans* bioassays, and intelligent sensory analysis (electronic nose (E-nose), electronic tongue (E-tongue), and gas chromatography-ion mobility spectrometry (GC-IMS)). Results indicated that delaying harvest to 15 September significantly promoted bioactive accumulation, with total saponins reaching 9.54 g kg^−1^ at this stage. Stir-frying the optimal raw material further enhanced pharmacological efficacy; spinosin content increased 1.48-fold, and *C. elegans* motility cessation time significantly shortened from 240 s to 180 s, demonstrating superior sedative activity. Additionally, stir-frying improved the total sensory score from 53.8 to 80.4, characterized by a harmonized balance of bitterness and umami. GC-IMS analysis identified Maillard reaction products, specifically 2-methylpyrazine and 2-methylbutanal as key markers responsible for the distinctive roasted aroma. Consequently, harvesting the fruits of *Ziziphus jujuba* var. *spinosa* at physiological maturity, followed by the stir-frying of ZSS effectively enhances its sedative effects and flavor profile.

## 1. Introduction

*Ziziphi spinosae semen* (ZSS) comprises the dried mature seeds of Chinese wild jujube (*Ziziphus jujuba* Mill. var. *spinosa* (Bunge) Hu ex H. F. Chow) [1], is a traditional herbal medicine widely prescribed for insomnia and anxiety [2]. Due to its excellent safety profile and therapeutic efficacy, ZSS is officially recognized as a medicine-food dual-purpose resource in China [3]. Its therapeutic efficacy is primarily attributed to multiple bioactive constituents, including flavonoids (e.g., Spinosin), saponins (e.g., Jujuboside A and B), and alkaloid as well as polyphenolic compounds, vitamins (e.g., vitamin C), and essential fatty acids [4]. Modern pharmacological studies confirm that saponins and flavonoids play crucial roles in regulating the central nervous system [5] and exerting antioxidant activity [6]. The accumulation of these bioactive components is a dynamic process influenced by the optimal harvest date, which impacts the plant’s metabolism and ultimately dictates its phytochemical profile [7]. Studies on crops like *Ginseng* [8] and *Hemerocallis citrina* [9] indicate that optimal harvest timing is critical for maximizing target secondary metabolite yield. Furthermore, pre-harvest factors, primarily cultivation environments, jointly influence this maturation process, which is accompanied by significant changes in botanical and physical traits such as seed color (shifting from pale white to reddish-brown), shape (ovoid development), and mechanical properties (e.g., shell hardness), all of which serve as crucial indicators for determining the ideal window for pharmaceutical collection.

The optimal harvesting period for ZSS remains underexplored, lacking systematic studies to determine the maturation stage for maximal bioactive compound accumulation. This knowledge gap has significant practical implications. Notably, driven by the ZSS’s high market value, a prevalent practice known as “green harvesting” has emerged, where collection begins as early as 15 July to capitalize on price advantages. However, the impact of this premature harvest on the final quality and pharmacological potency of the ZSS constitutes a critical, unaddressed research question. It is hypothesized that early harvesting may compromise the development of key functional constituents, yet this remains scientifically unverified.

Furthermore, the Chinese Pharmacopoeia stipulates that ZSS must be stir-fried prior to use, implying that processing enhances therapeutic properties [10]. However, the effects of stir-frying on the functional components are poorly characterized. Additionally, as a medicinal food homology product, the sensory attributes (taste and aroma) of ZSS are crucial for consumer acceptance and compliance, indirectly influencing its efficacy in dietary regimens.

Comprehensive research is therefore essential to establish science-based guidelines for harvesting and processing, ensuring the final product delivers consistent bioactivity, quality, and desirable organoleptic properties. This study aims to investigate different harvest times and stir-frying influence the functional compounds, pharmacological activities, as well as the sensory profiles of ZSS. The findings are expected to provide practical guidance for the optimized utilization of this medicinal food homology product.

## 2. Results

### 2.1. Influence of Harvest Time on Bioactive Compounds and Efficacy of ZSS

**Figure 1:**
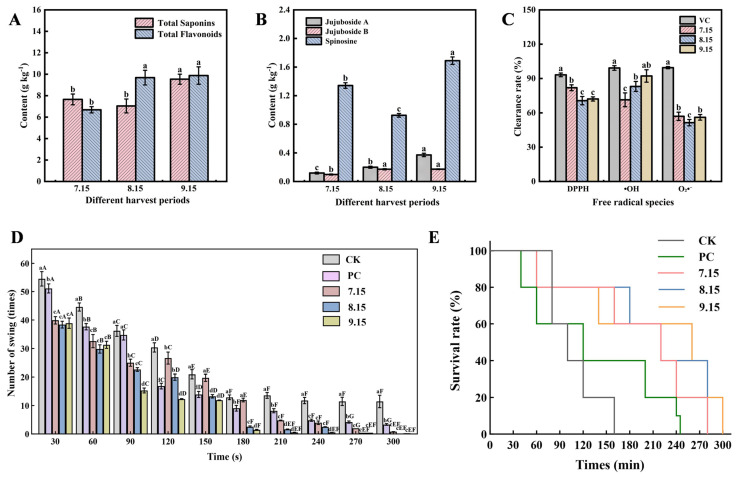
Changes in the Content of Bioactive Components in *Ziziphi Spinosae Semen* at different harvesting times. (**A**) Total saponins and total flavonoids content. (**B**) Contents of jujuboside A, jujuboside B and spinosin. (**C**) In vitro antioxidant capacity of *Ziziphi Spinosae Semen*. (**D**) In vivo sedative effect evaluated by body bends of *C. elegans*. (**E**) In vivo antioxidant activity assessed by survival rate of *C. elegans* under oxidative stress. Note: 7.15, 8.15, and 9.15 represent the harvest dates of 15 July (Green-ripe stage), 15 August (White-ripe stage), and 15 September (Fully mature stage), respectively. CK indicates the control group. Data are presented as the mean ± standard error from three independent biological replicates. Different lowercase letters (e.g., a, b, c, d) indicate significant differences (*p* < 0.05) among different treatments within the same parameter or at the same time point. In (**D**), different uppercase letters (e.g., A, B, C, D, E, F, G) indicate significant differences (*p* < 0.05) within the same treatment group across different time points. Statistical significance was determined by one-way analysis of variance (ANOVA), followed by Tukey’s test.

The initial harvest (15 July) yielded the lowest levels of both total flavonoids and total saponins. With delayed harvesting, total flavonoid content increased progressively, stabilizing after 15 August. In parallel, the total saponins content first slightly decreased and then increased, reaching its peak concentration (9.54 g kg^−1^) in the final harvest (15 September). Consequently, the levels of these two components converged at full maturity, with the final saponin content being marginally lower than that of flavonoids (Figure 1A).

In addition to the analysis of total saponins and total flavonoid, we measured the levels of three key sedative compounds: jujuboside A, jujuboside B, and spinosin, in samples from three harvest times (Figure 1B). The content of jujuboside A showed a progressive increase with later harvest time, culminating in a concentration approximately 1.7-fold higher in the 15 September samples compared to those from 15 July. Jujuboside B content also increased initially but plateaued after 15 August. In contrast, spinosin exhibited a more complex pattern, decreasing by mid-August before rising sharply to its peak level of 1.86 g kg^−1^ on 15 September. Collectively, these results demonstrate a clear trend of enhanced accumulation of these principal sedative constituents with delayed harvest time.

The in vitro antioxidant activities of ZSS harvested at different time points were systematically evaluated by measuring their scavenging capacities against DPPH, hydroxyl, and superoxide anion radicals (Figure 1C). Extracts from ZSS harvested on 15 August and 15 September displayed similar DPPH radical-scavenging capacities. In contrast, the extract obtained on 15 July demonstrated the highest activity Notably, the 15 September harvest demonstrated higher scavenging capacity against both hydroxyl and superoxide anion radicals. Specifically, its hydroxyl radical scavenging rate reached 92.79%, which was significantly higher than those observed in samples from other harvest times.

Subsequently, we evaluated the functional sedative and in vivo antioxidant effects of these compositional changes using a *Caenorhabditis elegans* model. Regarding sedative activity, recording the number of body bends within 30-s intervals revealed that all extract-treated groups exhibited decreased motility following an initial surge in activity (Figure 1D). With the 15 September harvest exhibiting the fastest and strongest inhibitory effect. At the 90-s mark, nematodes treated with the 15 September extract exhibited a significantly lower number of body bends compared to other groups, and their movement ceased entirely by 180 s. This was significantly earlier than the cessation observed with the 15 July and 15 August samples at 240 s, indicating a stronger sedative potential in the later harvest. Regarding in vivo antioxidant effects, multiple bioactive components in ZSS effectively improved the survival of nematodes under oxidative stress, potentially by modulating the antioxidant defense system (Figure 1E). Specifically, the 15 July harvest sample extended nematode lifespan by approximately 120 min, while the 15 August and 15 September samples showed similar effects, each extending lifespan by about 140 min. This further demonstrates that later-harvested ZSS exhibit superior overall biological activity.

ZSS harvested on 15 July and 15 August contained significantly lower levels of key bioactive constituents compared to the 15 September harvest. This discrepancy is likely due to seed immaturity from premature harvesting. The shorter growth period results in compromised development, leading to insufficient accumulation of nutrients and yielding underdeveloped, less plump kernels. Furthermore, the high moisture content associated with premature harvest may facilitate the growth of *Aspergillus flavus*, further deteriorating seed quality [11]. Therefore, harvest time management is a critical factor for ensuring ZSS quality. The present findings indicate that in Zanhuang, Hebei Province, the optimal harvest period for ZSS begins after 15 September.

### 2.2. Influence of Stir-Frying on Bioactive Compounds and Efficacy of ZSS

Based on the comparative assessment of harvest timing, ZSS harvested on 15 September was identified as the optimal raw material and selected for subsequent stir-frying experiments. The impact of thermal processing was then systematically evaluated by contrasting the bioactive composition, sedative-hypnotic activity, and antioxidant capacity between the raw and stir-fried samples.

**Figure 2:**
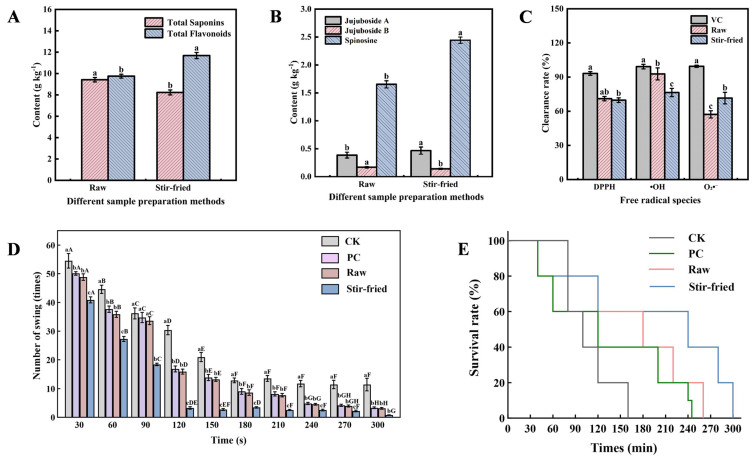
Changes in the content of bioactive components in *Ziziphi Spinosae Semen* before and after stir-frying. (**A**) Total saponins and total flavonoids content. (**B**) Contents of jujuboside A, jujuboside B and spinosin. (**C**) In vitro antioxidant capacity of *Ziziphi Spinosae Semen*. (**D**) Sedative effect evaluated by body bends of *C. elegans.* (**E**) In vivo antioxidant activity assessed by survival rate of *C. elegans* under oxidative stress. Data are presented as the mean ± standard error from three independent biological replicates. Different lowercase letters (e.g., a, b, c) indicate significant differences (*p* < 0.05) among different treatments within the same parameter or at the same time point. In Figure 1D, different uppercase letters (e.g., A, B, C, D, E, F, G, H) indicate significant differences (*p* < 0.05) within the same treatment group across different time points. Statistical significance was determined by one-way analysis of variance (ANOVA), followed by Tukey’s test.

As detailed in Figure 2A, the content of total flavonoids increased significantly from 9.75 g kg^−1^ to 11.68 g kg^−1^ after stir-frying. Conversely, the total saponin content exhibited a significant decrease from 9.42 g kg^−1^ to 8.31 g kg^−1^. These findings demonstrate that the stir-frying process markedly influenced the levels of both compound categories in the seed.

The impact of stir-frying on the three key compounds was quantified by HPLC (Figure 2B). The process yielded a divergent response: jujuboside A exhibited a moderate increase, jujuboside B showed a slight decline, and spinosin rose sharply to 1.48 times its original value. Beyond these individual changes, the total content of sedative compounds was significantly elevated in stir-fried samples, suggesting a potentially enhanced therapeutic profile. This provides a chemical basis for the traditional method and demonstrates how processing enhances the overall bioactivity of the material.

The antioxidant capacity of raw and stir-fried ZSS were evaluated and compared with vitamin C (VC) as a reference (Figure 2C). Raw samples exhibited superior scavenging capacity against hydroxyl radicals, which was significantly higher than that for DPPH and superoxide anion radicals. The stir-frying process exerted divergent effects on antioxidant capacity: it preserved the DPPH scavenging activity and significantly enhanced the superoxide anion scavenging capacity but compromised the hydroxyl radical scavenging potential.

The functional sedative and antioxidant effects of ZSS were evaluated using a *Caenorhabditis elegans* model, comparing raw (Raw) and stir-fried (Stir-fried) ZSS extracts. A vehicle control (solvent only) was prepared in parallel to ensure that the sedative and antioxidant effects were solely attributable to the ZSS phytochemicals rather than the solvent.

Sedative activity was quantified by recording the number of body bends within 30-s intervals. Both Raw and Stir-fried ZSS extract-treated groups exhibited a significant decrease in motility following an initial activity surge, in contrast to the control (CK) group (Figure 2D). Notably, the Stir-fried ZSS group demonstrated a superior inhibitory effect, with nematode motility reaching its lowest level within 90 s and ceasing entirely by 180 s. This was significantly faster than the Raw ZSS group, which required 240 s to achieve complete immobility, indicating that the stir-frying process enhances the sedative potential of ZSS.

Concurrently, the antioxidant effect was assessed by measuring the survival rate under oxidative stress (Figure 2E). Both ZSS treatments significantly extended the nematode lifespan compared to the CK group. The Stir-fried ZSS treatment provided the most robust protection, extending the mean lifespan by approximately 140 min. The raw ZSS treatment also exhibited a considerable effect, prolonging the lifespan by about 120 min. These results demonstrate that the bioactive components in ZSS effectively improve the in vivo antioxidant capacity of nematodes, with the stir-fried product exhibiting superior protective effects against oxidative stress.

In summary, the processing method critically influences the bioactivity of ZSS, with the stir-fried sample exhibiting superior overall efficacy in both sedative and antioxidant assays.

### 2.3. Sensory Evaluation Analysis

**Figure 3:**
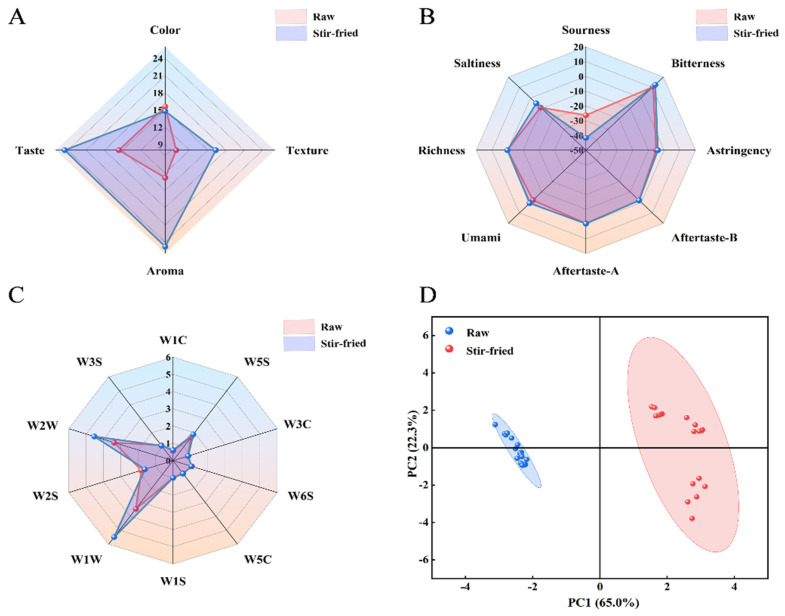
Sensory evaluation of raw and stir-fried *Ziziphi Spinosae Semen*. (**A**) Sensory evaluation scores. (**B**) E-tongue taste attribute radar chart. (**C**) E-nose sensor response radar chart. (**D**) The scatter plot of principal component analysis.

After stir-frying ZSS, significant differences were observed in sensory scores (Figure 3A). Both raw and stir-fried samples were evaluated across four dimensions: color (20 points), texture (20 points), aroma (30 points), and flavor (30 points). Except for color, where raw samples scored slightly higher than Stir-fried ones, the Stir-fried samples significantly outperformed the raw samples in the other three evaluation criteria (texture, aroma, and flavor). The total sensory evaluation scores showed raw samples at 53.8 points and Stir-fried samples at 80.4 points. Therefore, stir-frying significantly enhances the sensory evaluation of ZSS.

E-tongue analysis revealed that stir-frying significantly altered the taste profile of ZSS (Figure 3B). Compared to the raw sample, stir-frying markedly reduced sourness, while significantly enhancing bitterness and umami. Astringency and saltiness were also moderated to a certain extent, with aftertaste characteristics remaining stable. These collective changes demonstrate that the stir-frying process effectively harmonizes the taste of ZSS, resulting in a more mellow and balanced sensory experience.

Radar chart analysis of the electronic nose indicated that the sensors contributing most significantly to sample differentiation were W1W (sensitive to sulfides), W2W (sensitive to organic sulfur compounds), and W2S (sensitive to alcohols, aldehydes, and ketones) (Figure 3C). The response values of W1W and W2W sensors were significantly enhanced in the stir-fried product, while the W2S response value was slightly higher in the raw product. This indicates that the stir-frying process promotes the formation of sulfur-containing compounds while reducing some oxygen-containing compounds.

The principal component analysis (PCA) analysis results of raw and stir-fried ZSS based on the signals from the electronic nose sensor (Figure 3D). The variance contributions of its two principal components are 65.0% and 22.3%, respectively, accounting for a total of 87.3% of the valid information. This effectively captures nearly all sample information and highlights the differences between them. The raw and stir-fried samples are clearly separated.

### 2.4. Analysis of Volatile Organic Compounds in Stir-Fried ZSS

**Figure 4:**
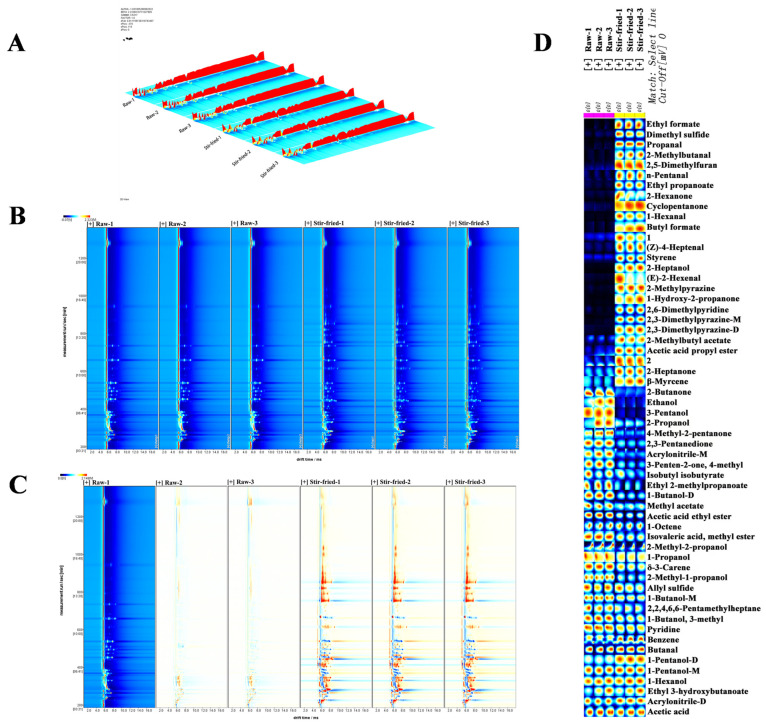
Volatile compounds of *Ziziphi Spinosae Semen* across stir-fried and raw. (**A**) Three-dimensional topographic map; (**B**) topographic map; (**C**) sample differences comparison map. Plot based on retention time (s), relative ion travelling time (a.u.), and peak intensity (V) in the data; (**D**) Fingerprints of volatile compounds in stir-fried and raw *Ziziphi Spinosae Semen*. Note: For visual clarity, concise common names are used in this figure. Please refer to Supplementary Table S2 for the corresponding strict IUPAC nomenclature of all identified volatile organic compound (VOCs).

To delineate the impact of stir-frying on the volatile profile, the VOC compositions of the samples before and after stir-frying were analyzed using GC-IMS. For a direct visualization of the resulting changes, the fingerprint of the raw sample was set as the reference, against which the stir-fried sample was directly compared, obtain differential comparison diagrams. The three-dimensional topographic profiles of volatile organic compounds (VOCs) in ZSS samples before and after stir-frying, revealing distinct differences in peak intensities (Figure 4A).

A two-dimensional top-view visualization of ZSS volatiles is presented. In this plot, the vertical and horizontal axes correspond to gas chromatography retention time and ion mobility drift time, respectively, with the red line at 1.0 indicating the normalized RIP (Reaction Ion Peak) (Figure 4B). The spots with varying shades and sizes represent the VOC detection results, whiter spots indicate lower volatile content, while redder spots correspond to higher concentrations.

In the comparison chart of the differences between the raw sample and the stir-fried sample, the redder the dot, the higher the content compared to the control group, while the bluer the dot, the lower the content (Figure 4C). The white ones cancel each other out. From this, it can be observed the detected VOCs are mainly concentrated within the retention time range of 200 to 600 s, and the drift time range is 1.0 to 1.6 ms. A large number of red dots can be observed in the chromatogram of the stir-fried samples, confirming that the concentration of some volatile compounds has significantly increased after the stir-frying treatment.

For further analysis, a fingerprint chromatogram was produced (Figure 4D). The results of the library search qualitative analysis for the samples are shown in Table S2. In total, 58 signal peaks were detected, from which 54 volatile compounds were identified. Comprising 10 esters, 12 alcohols, 4 pyrazines, 8 ketones, 6 aldehydes, 4 hydrocarbons, 2 monoterpenes and 8 other substances. The formation of dimers or multimers was caused by higher proton affinities or higher concentrations, some compounds like 1-pentanol, 1-butanol, 2,3-dimethylpyrazine, acrylonitrile can generate multiple signals at varying concentrations, such as in the form of monomers, dimers, or trimers. To avoid duplicate counting, the multiple signals (e.g., monomers and dimers) corresponding to a single compound were consolidated and reported as one unique volatile entity.

The contents of 26 aroma components, including butyl formate (fruity flavors, including plum, rum and brandy), acetic acid ethyl ester (aroma of wine), 1-hydroxy-2-propanone (caramel aroma), (E)-2-hexenal (cheese flavor), 2-heptanol (fresh lemon flavor, sweet, floral and fruity), 2-methylpyrazine (nutty flavor, baking aroma), 2-methylbutanal (malt aroma, chocolate aroma, fruity aroma and nutty background), have significantly increased. Meanwhile, the contents of 16 aroma components were significantly reduced, such as 1- butanol (strong alcohol smell), 2-propanol (pungent alcohol smell and bitter taste), 2,3-pentanedione (spicy, buttery). It is worth noting that after being stir-fried, the relative contents of 2-methylpyrazine, 2-methylbutyraldehyde and ethyl acetate in stir-fried ZSS samples are ten times those in raw ZSS samples. These three compounds respectively have strong nutty, coffee and fruit aromas. Research has proved [12] that ethyl acetate also has a wine-like aroma.

**Figure 5:**
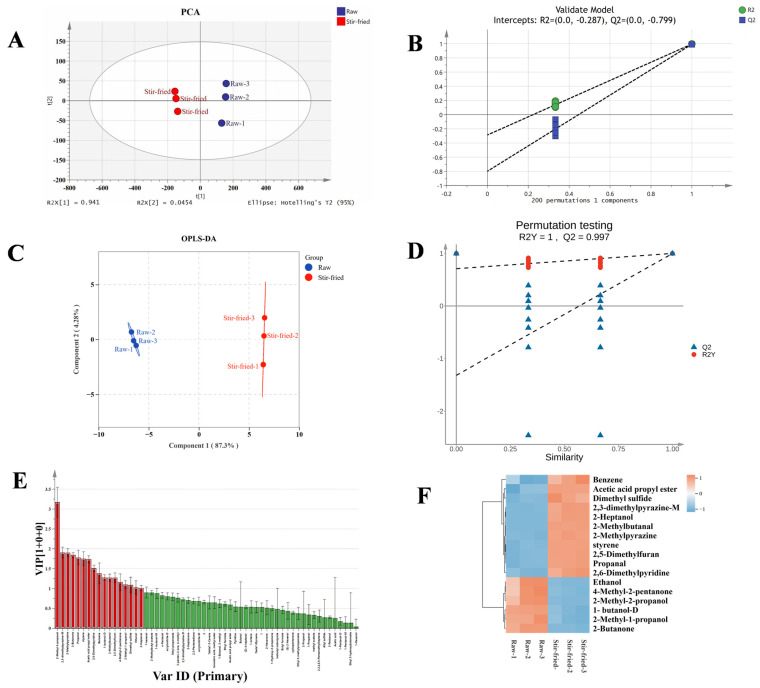
Orthogonal partial least squares discriminant analysis (OPLS-DA), PCA analysis of *Ziziphi Spinosae Semen* across stir-fried and raw using GC-IMS. (**A**) Plot of PCA scores; (**B**) plot of cross-validation of the 200-substitution test; (**C**) Plot of OPLS-DA scores; (**D**) Plot of cross-validation of the 200-substitution test; (**E**) The red section represents the key differentiated compounds with variable importance in projection (VIP) > 1; (**F**) VIP > 1 Correlation Heatmap. Note: For visual clarity, concise common names are used in this figure. Please refer to Supplementary Table S2 for the corresponding strict IUPAC nomenclature of all identified VOCs.

PCA was employed to visualize the natural clustering and intrinsic variation among the raw and stir-fried ZSS samples. The score plot (Figure 5A) revealed a clear separation between the two groups along the principal components. It shows a clear separation between the two groups. The raw samples are in quadrants 1 and 4, while the stir-fried samples are in quadrants 2 and 3.

The key findings from the PCA model are as follows: The clear separation between raw and stir-fried sample clusters. Model robustness was verified through 200 permutation tests, where the Q^2^ regression line below zero (R^2^ = −0.287, Q^2^ = −0.799) in Figure 5B confirmed reliability without overfitting.

To accurately identify the significantly altered volatile compounds, OPLS-DA was employed to differentiate observation groups and identify key variables responsible for intergroup differences (Figure 5C). As shown in Figure 5D, the samples exhibit excellent separation. The variance contributions of its two principal components are 87.3% and 4.28%, respectively, accounting for a total of 91.58% of the valid information. Model robustness was verified through 200 permutation tests, confirmed reliability without overfitting.

The contribution of individual aroma compounds to the overall profile of ZSS samples was evaluated using VIP (Variable Importance in Projection) values. As depicted in Figure 5E, 17 aroma components with VIP values greater than 1 were identified as significant contributors: 2-methyl-2-propanol, 2,3-dimethylpyrazine-M, 2-methylpyrazine, 2-butanone, propanal, styrene, acetic acid ethyl ester, 2,6-dimethylpyridine, benzene, 1-butanol-D, 2-methylbutanal, 2,5-dimethylfuran, 4-hethyl-2-pentanone, 2-methy1-1-propanol, 2-methy1-1-propanol, ethanol, 2-heptanol. These compounds are considered the primary volatile substances responsible for the distinctive aroma of ZSS.

Acetic acid propyl ester, 2-heptanol, 2,3-dimethylpyrazine-M, 2-methylpyrazine, 2, 6-dimethylpyridine, 2-methylbutanal, propanal, benzene, styrene, 2, 5-dimethylfuran, dimethyl sulfide were positively correlated with the stir-fried samples and negatively correlated with the raw samples 1-butanol-D, 2-methyl-1-propanol, ethanol, 4-methyl-2-pentanone, and 2-butanone were negative correlated with the stir-fried samples and positively correlated with the raw samples.

As shown in Figure 5F, 17 volatile compounds effectively distinguished the differences between samples. In summary, the reliable discrimination between raw and stir-fried ZSS can be achieved by screening specific volatile marker compounds in combination with multivariate statistical analyses, such as PCA and cluster analysis. While these VOCs characterize the distinctive aroma profile of processed ZSS, their specific contribution to the observed biological activities remains to be elucidated, as they may primarily serve as indicators of the degree of processing.

## 3. Discussion

This study systematically investigated the effects of harvest timing and stir-fried process on the content of major bioactive components and antioxidant activity in ZSS. Results indicate that appropriately delayed harvesting significantly promotes the accumulation of multiple bioactive components in ZSS, including total flavonoids, total saponins, Jujuboside A, Jujuboside B, and Spinosin. The content of each component peaked when harvested on 15 September. This accumulation pattern synchronized with fruit maturation, consistent with the secondary metabolism patterns of most medicinal plants [13], where a longer growth period provides essential conditions for the biosynthesis and storage of bioactive compounds [14]. However, the 15 July harvest demonstrated the highest DPPH radical scavenging activity despite its lower flavonoid and saponin content (Figure 2C). This counterintuitive peak may be attributed to unmeasured bioactive constituents, such as procyanidins or phenolic acids, which often reach maximum concentrations in immature seeds to protect the developing embryo from oxidative stress [15]. Correspondingly, extracts from late-harvested and stir-frying exhibited stronger sedative and sleep-promoting activity in the *Caenorhabditis elegans* model, potentially related to increased spinosin and saponin content [4]. Furthermore, the content of Jujuboside A, Jujuboside B, and Spinosin showed a significant positive correlation with the sedative effect in nematodes, further confirming the central role of these components in the hypnotic efficacy of ZSS [16].

The changes in chemical composition during stir-frying are influenced by multiple interacting factors. While the high temperature may cause the degradation of certain thermally unstable compounds, it also physically disrupts the plant cell walls as the seeds expand and become crisp. This structural breakdown significantly enhances the solvent penetration and the subsequent release and extractability of intracellular bound flavonoids and saponins. [17]. Additionally, thermal processing may trigger complex chemical transformations, collectively contributing to the altered chemical profile observed in stir-fried ZSS.

Sensory evaluation, e-nose, and e-tongue analyses collectively indicate that stir-fried treatment significantly enhances the overall sensory quality of ZSS. Sensory evaluation revealed that stir-fried samples achieved a total score of 80.4 points, markedly higher than raw samples, with particularly notable improvements in texture, aroma, and flavor [18]. This may be attributed to a slight decrease in color score due to heat-induced browning reaction [19]. Electronic tongue analysis further revealed that stir-frying significantly intensified the bitterness and umami of ZSS while markedly reducing sourness. The decrease in sourness may relate to the volatilization or thermal degradation of short-chain organic acids during heating [20]. Concurrently, the enhancement of bitterness and umami likely stems from the release or transformation of flavor compounds such as alkaloids and peptides during heating [21]. Electronic nose detection further confirmed aroma changes, with significantly enhanced responses from W1W (sensitive to sulfides) and W2W (sensitive to organic sulfides) sensors in the stir-fried samples. This indicates increased formation of sulfur-containing flavor compounds [22], closely related to the development of stir-fried and nutty aroma [23]. Concurrently, the W2S sensor (sensitive to alcohols, aldehydes, and ketones) exhibited reduced response, suggesting that some oxygenated volatiles may decompose or participate in flavor restructuring during stir-frying [24].

Through in-depth analysis of VOCs via GC-IMS, this study investigates the specific effects of the stir-frying process on the aromatic components of ZSS. Differential comparison spectra clearly demonstrates that stir-frying treatment induces significant alterations in VOC composition, with concentrations of multiple volatile components markedly increasing post stir-frying. Key compounds such as 2-methylpyrazine, 2,3-dimethylpyrazine, 2-methylbutanal, and 2,6-dimethylpyridine showed extremely significant positive correlations with the stir-fried samples. These compounds are widely recognized as classic products of the Maillard reaction. Their marked increase clearly indicates that stir-frying is an effective pathway for generating baked [25], nutty [25], and coffee-like aromas [26] in ZSS. The concurrent elevation of esters like ethyl acetate and propyl acetate contributes desirable fruity [27] and wine-like notes [28], enhancing the overall aroma complexity and richness [29]. Furthermore, increases in 2-heptanol (contributing fresh, sweet nuances) [30] and sulfur compounds like dimethyl sulfide may enhance the depth and complexity of stir-fried aromas [31]. Conversely, the negative correlation of compounds such as 1-butanol, 2-methyl-1-propanol, ethanol, and 2-butanone with stir-fried samples is equally significant. These short-chain alcohols and ketones are typically associated with pungent, alcoholic, and green sensory characteristics [32]. Their significant reduction during stir-frying suggests they may serve as precursors consumed in the Maillard reaction or volatilize due to heating, thereby optimizing the aroma by eliminating undesirable off flavors. This dual effect of generating favorable aromas and reducing unfavorable ones collectively creates an exceptional and balanced sensory experience. These results align with the findings of Wang et al. [33], who reported that thermal processing significantly enhances the extractability of bioactive constituents like flavonoids in ZSS. While they macroscopically observed the emergence of a distinct aroma during frying, our GC-IMS analysis further provides a molecular basis for this by identifying specific Maillard reaction products, such as 2-methylpyrazine and 2-methylbutanal.

It is worth noting that while strictly defined cultivation under Good Agricultural Practices (GAP) is the ideal model for pharmaceutical standardization, a substantial portion of the current ZSS supply is still sourced from semi-wild habitats within traditional authentic regions. In these natural environments, plants are frequently exposed to various environmental stressors, which have been shown to trigger the biosynthesis of key secondary metabolites like saponins and flavonoids. Therefore, in the absence of fully controlled cultivation, identifying the optimal harvest date based on physiological maturity serves as a vital and pragmatic quality-control strategy. This ensures that the raw materials collected from diverse natural habitats maintain the high potency and consistency required for pharmaceutical applications. Although this study systematically elucidated the regulatory effects of harvest timing and stir-frying process on the active components and pharmacological efficacy of ZSS, as well as the influence of stir-frying on flavor quality, certain limitations remain. All samples were sourced from a single production area in Zanhuang, Hebei Province, in 2024, which may not fully reflect the impact of diverse geographical origins and interannual climatic fluctuations on component accumulation and aroma formation. Future multi-regional and multi-year studies are warranted to further validate the stability of these harvest recommendations. Furthermore, as the characteristic red color of the mature fruit suggests the potential accumulation of anthocyanins during the ripening process, the dynamic changes of these compounds are of great physiological interest. Although the current study primarily focused on the core pharmacodynamic components of ZSS (such as saponins, flavonoids, and spinosin) to align with pharmacopoeial standards, profiling the anthocyanin content and its correlation with visual color changes remains a valuable subject. Therefore, a targeted quantitative analysis of anthocyanins will be incorporated into our future research to provide a more comprehensive understanding of the ripening-induced metabolic shifts in the plant.

## 4. Materials and Methods

### 4.1. Plant Materials and Treatments

**Figure 6:**
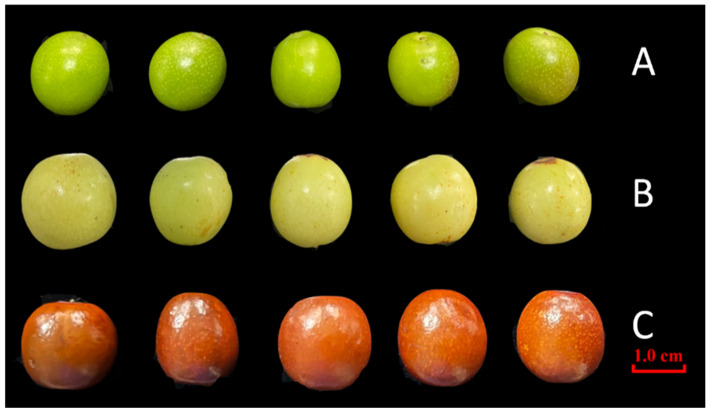
Phenotypic images of wild jujube (*Ziziphus jujuba* Mill. var. *spinosa* (Bunge) Hu ex H. F. Chow) at three developmental stages: (**A**) Green-ripe stage (15 July 2024); (**B**) White-ripe stage (15 August 2024); (**C**) Mature stage (15 September 2024). Scale bar = 1.0 cm.

The fruits of wild jujube *(Ziziphus jujuba* Mill. var. *spinosa* (Bunge) Hu ex H. F. Chow) plants were collected from wild habitats in Zanhuang County, Hebei Province, China. Sampling was conducted across three distinct maturation stages in 2024: “Green-ripe” (15 July), “White-ripe” (15 August), and “Fully mature” (15 September). The maturity stages were determined based on standardized phenotypic indicators: the ‘Early’ stage was characterized by solid green skin; the ‘Mid’ stage by a transition to pale white-green; and the ‘Late’ stage by a saturated deep-red color covering the entire fruit (Figure 6). All harvested samples were authenticated by Professor Mengjun Liu from Hebei Agricultural University. At each time point, three biological replicates (approximately 1000 fruits per replicate) were systematically collected. Immediately after harvest, the fruits were transported to the laboratory at Hebei Agricultural University. The seeds were then carefully separated from the pulp. Following separation, the seeds were washed and uniformly dried at 35 °C for 48 h until a constant weight was achieved. Subsequently, the dried seeds were sealed in bags and stored at −20 °C to preserve their chemical integrity until further analysis. The raw materials were authenticated as the dried mature seeds of *Ziziphus jujuba* Mill. var. *spinosa* (Bunge) Hu ex H.F. Chow by Mengjun Liu (Professor of Hebei Agricultural University).

### 4.2. Samples Preparation

#### 4.2.1. Preparation of ZSS and Stir-Fried ZSS

Samples harvested on 15 July and 15 August: The seeds were first pre-dried to a constant weight, then crushed and passed through a No. 4 sieve (65 mesh). The resulting powder was collected and analyzed immediately.

Samples harvested on 15 September: The samples were randomly divided into two groups. The kernels were carefully cracked to obtain intact seeds.

Raw group: One group remained unprocessed and served as the raw seeds.

Stir-fried group: The other group was stir-fried according to the general processing guidelines in the Pharmacopoeia of the People’s Republic of China (2020 edition) [34] until the seeds became expanded and slightly darkened in color.

After cooling to room temperature, both the raw and stir-fried seeds were pre-dried to a constant weight to rigorously eliminate the interference of varying moisture contents. Subsequently, they were crushed and passed through a No. 4 sieve (65 mesh). The resulting powders were separately collected, placed in sealed containers, and stored at −20 °C for subsequent analysis. Prior to each analysis, the sealed samples were allowed to equilibrate to room temperature in a desiccator to prevent moisture condensation. Consequently, all quantitative data in our study are strictly calculated and expressed on a dry weight (DW) basis.

#### 4.2.2. Sample Preparation for Total Flavonoids, Saponins, Jujuboside A, Jujuboside B, and Spinosin Analysis

The ZSS powder was degreased according to the method described by Zhang et al. [35]. Subsequently, 1.5 g of degreased ZSS powder was subjected to ultrasound-assisted extraction using 30 mL of 95% (*v*/*v*) ethanol for 50 min. The mixture was allowed to stand for 30 min and centrifuged at 9400 rpm (Sorvall ST 8R, Thermo Fisher Scientific (China) Co., Ltd., Shanghai, China). The supernatant was then collected, with one portion used for the determination of total flavonoids and saponins, while another portion was concentrated to dryness at 45 °C using a rotary evaporator (RV 10, IKA^®^-Werke GmbH & Co. KG, Staufen, Germany). The dried residue was then redissolved in 3 mL of methanol in a centrifuge tube and filtered through a 0.22 μm membrane filter to ensure purity and remove particulate matter for subsequent analysis of jujuboside A, jujuboside B, and spinosin.

### 4.3. Analysis of Total Saponins and Total Flavonoids

Preparation of Reference Standards: A jujuboside A standard stock solution (0.20 mg/mL) was prepared by dissolving 5.00 mg (accurately weighed) of jujuboside A reference standard in methanol, transferring to a 25 mL volumetric flask, and diluting to volume with methanol. Similarly, a rutin standard stock solution (0.20 mg/mL) was prepared by dissolving 10.00 mg of rutin reference standard in methanol within a 50 mL volumetric flask and diluting to the mark with methanol.

Determination of Total Saponins: The total saponins content was quantified at a detection wavelength of 544 nm using the vanillin-perchloric acid colorimetric method described by Liu et al. [36]. with jujuboside A as the reference standard.

Determination of Total Flavonoids: The total flavonoids content was quantified at a detection wavelength of 510 nm using the colorimetric method described by Belonio et al. [37], using rutin as the standard.

Reference standards of jujuboside A (Cat. No. B21224), jujuboside B (Cat. No. B21225), spinosin (Cat. No. B21200), and rutin (Cat. No. B25342) were purchased from Shanghai Yuanye Bio-Technology Co., Ltd. (Shanghai, China).

### 4.4. Quantitative Analysis of Jujuboside A, Jujuboside B, and Spinosin by HPLC

Reference standards of jujuboside A, jujuboside B, and spinosin (purity ≥ 98%, purchased from Shanghai Yuanye Bio-Technology Co., Ltd., Shanghai, China) were dissolved in methanol to prepare stock solutions (0.2 mg/mL). HPLC analysis was performed on an Agilent 1260 Infinity system (Agilent Technologies, Santa Clara, CA, USA) equipped with a Diode Array Detector (DAD). Separation was achieved on a Diamonsil C18 column (250 mm × 4.6 mm, 5 μm). The injection volume was 10 μL, and the flow rate was maintained at 0.8 mL/min, with a total detection time of 25 min.

The detection conditions were optimized for each analyte:

For jujubosides A and B: An isocratic elution was applied, where the mobile phase consisted of acetonitrile and 0.1% aqueous acetic acid (35:65, *v*/*v*). The column temperature was 30 °C, and detection was monitored at 204 nm.

For spinosin: An isocratic elution was also employed, with the mobile phase was acetonitrile and 0.1% aqueous acetic acid (20:80, *v*/*v*). The column temperature was 25 °C, with detection at 335 nm.

The preparation of the reference solution, with 1 mg each of jujuboside A, jujuboside B, and spinosin, accurately weighed, was added into 5 mL volumetric flasks, respectively, with the volume fixed to the scale line with methanol, prepared with 0.2 mg/mL of standard solution, diluted into solutions of different concentrations for standby, and injected in triplicates under the chromatographic conditions mentioned before.

For quantitative analysis, an external standard method was employed without the use of an internal standard. Calibration curves were constructed by plotting the peak areas against the corresponding analyte concentrations using linear regression. The analytical method exhibited excellent linearity for all targeted analytes within their tested concentration ranges, with correlation coefficients R^2^ ≥ 0.99. The exact contents of jujuboside A, jujuboside B, and spinosin in the samples were calculated based on these established regression equations.

### 4.5. In Vitro Antioxidant Activity Assay

The in vitro antioxidant capacity of ZSS extracts was systematically evaluated by assessing their radical scavenging activity against DPPH, hydroxyl, and superoxide anion radicals, using VC as a reference standard in all assays. To ensure a rigorous comparative analysis, both the ZSS extract sample solutions and the VC standard solutions were prepared at an identical concentration of 1.0 mg/mL (dissolved in ethanol). The DPPH free radical scavenging assay was performed according to Li et al.’s method with minor modifications [38]. 1 mL of sample solution was mixed with 2 mL of 0.2 mM DPPH ethanol solution, vigorously vortexed, and incubated in darkness at room temperature for 30 min before measuring the absorbance at 517 nm (TU-1810, Beijing Purkinje GENERAL Instrument Co., Ltd., Beijing, China). Where A_0_ represents the control absorbance (DPPH solution with ethanol), A_1_ denotes the sample mixture absorbance (sample with DPPH solution), and A_2_ corresponds to the sample background absorbance (sample with ethanol instead of DPPH).

Inhibition of DPPH radicals (%) = [A_0_ − (A_1_ − A_2_)]/A_0_ × 100 (1)

The hydroxyl radical scavenging assay was performed according to the method of Cheng et al. [39], Briefly, 0.2 mL of the sample extract was mixed with 1 mL of FeSO_4_ (6 mmol/L) and 1 mL of H_2_O_2_ (6 mmol/L), vortexed for 1 min, and incubated at 25 °C for 10 min. Then, 1 mL of salicylic acid solution (6 mmol/L) was added, vortexed, and incubated for 60 min. The absorbance was measured at 510 nm, recorded as A_1_. A blank control (A_0_) was prepared by substituting distilled water for the sample extract. A negative control (A_2_) was prepared by substituting distilled water for H_2_O_2_ while keeping the sample extract and other reagents. The hydroxyl radical scavenging rate was calculated as:

Hydroxyl radical scavenging rate (%) = (A_0_ − A_1_ + A_2_)/A_0_ × 100 (2)

The superoxide anion radical scavenging activity was evaluated according to the method described by Goto et al. [40], using a non-enzymatic NADH/PMS/NBT system. Briefly, 0.01 mL of the sample extract was reacted with 0.05 mL of 250 mmol L^−1^ phosphate buffer (pH 7.2), 0.025 mL of 2 mmol L^−1^ NADH, and 0.025 mL of 0.5 mmol L^−1^ NBT. Then, 0.025 mL of 0.03 mmol L^−1^ PMS was added to initiate the reaction. After vortexing and incubation for 5 min at 25 °C, the absorbance of the reduced formazan was measured at 560 nm (denoted as A_1_). A blank control (A_0_) used distilled water instead of the sample extract, and a negative control (A_2_) replaced PMS with distilled water.

Superoxide anion radical scavenging rate (%) = (A_0_ − A_1_ + A_2_)/A_0_ × 100 (3)

### 4.6. Evaluation of the Sedative-Hypnotic and Antioxidant Activities of Caenorhabditis elegans

#### 4.6.1. *C. elegans* Culture and Synchronization

Nematode culture and maintenance were conducted according to the method described by Ferris et al. [41], with slight modifications. Briefly, the wild-type *Caenorhabditis elegans* strain N2 was used in this study. The nematodes were maintained on Nematode Growth Medium (NGM) agar plates seeded with *Escherichia coli* OP50 as a food source and cultured at 20 °C. To obtain synchronized populations for the experiments, gravid adults were washed from the plates and treated with a bleaching solution (0.5 M NaOH and 5% NaClO) to release the eggs. The collected eggs were then incubated in M9 buffer at 20 °C overnight to hatch into synchronized L1 larvae, which were subsequently transferred to fresh NGM plates for further growth to the L4 stage. The synchronized L4 larvae were then collected for subsequent oxidative stress and sedative activity assays.

#### 4.6.2. Sample Treatment

Sample treatment was performed according to the method described by Kim et al. [42], with slight modifications. Briefly, the ZSS extract powders were dissolved in 70% (*v*/*v*) ethanol to prepare stock solutions. These solutions were then added to the surface of the NGM plates to achieve the designated test concentration of 5 mg/mL. Crucially, the final concentration of ethanol in the culture medium was strictly maintained below 0.5% (*v*/*v*) to eliminate potential solvent toxicity. A control group (serving as the negative control, CK) containing an equivalent volume of solvent was prepared in parallel.

#### 4.6.3. In Vivo Sedative Activity Assay

The in vivo sedative activity assay was conducted according to the method described by Kim et al. [42], with slight modifications. Briefly, synchronized L4 larvae were transferred to NGM plates containing ZSS extracts (5 mg/mL), the negative control (vehicle only), or a positive control (10 mM melatonin). After 24 h of incubation, The frequency of spontaneous body bends was then quantified during a 30 s interval within a total monitoring window of 300 s. A body bend was defined as a complete sinusoidal wave corresponding to the long axis of the body. Mechanical stimulation was employed only at the end of the observation to distinguish between sedative-induced quiescence and paralysis or death; worms that failed to respond to physical touch were excluded from the sedative analysis.

#### 4.6.4. In Vivo Oxidative Stress Resistance Assay

The in vivo oxidative stress resistance assay was performed according to the method described by Kim et al. [42], with slight modifications. L4-stage larvae were pre-treated with ZSS extracts (5 mg/mL), the negative control (vehicle only), or a positive control (10 mM sodium ascorbate) for 24 h. Subsequently, the nematodes were exposed to 0.2% H_2_O_2_ to induce oxidative stress. The survival rate was monitored every 20 min until all negative control worms succumbed. The survival curves were plotted to evaluate the protective effects of ZSS against oxidative damage.

### 4.7. Sensory Evaluation

#### 4.7.1. Human Sensory Panel

Sensory evaluation was performed according to the methods described by Lin et al. [43] and Yao et al. [44], with minor modifications. A trained sensory panel consisting of 10 assessors (5 males and 5 females, aged 18–50) was selected from Hebei Agricultural University based on their sensory acuity. Prior to the formal evaluation, all panelists underwent training to familiarize themselves with the sensory descriptors of ZSS.

The evaluation was conducted in individual sensory booths at room temperature, with purified water provided as a palate cleanser between samples. The samples (raw and stir-fried) were presented in a randomized order. Panelists scored the samples using a 100-point scale across four attributes: color (0–20), texture (0–20), aroma (0–30), and flavor (0–30). The detailed scoring criteria are listed in Table S1. Ethical Statement: This study was approved by the Ethics Review Committee of Hebei Agricultural University (No. 2025150). All participants provided informed written consent.

#### 4.7.2. E-Nose Analysis

The electronic nose (E-nose) analysis was performed according to the method described by Tang et al., with minor modification [45]. A portable electronic nose (PEN3, AIRSENSE, Schwerin, Germany) equipped with 10 metal oxide semiconductor sensors was employed. Accurately weighed ZSS powder (1.0 g) was placed in a 20 mL headspace vial and equilibrated. The measurement parameters were set as follows: sample preparation time, 5 s; measurement time, 60 s; flush time, 60 s; and chamber flow rate, 400 mL/min. The sensor response data collected at the stable stage (roughly 48–52 s) were used for subsequent analysis.

#### 4.7.3. E-Tongue Analysis

The electronic tongue (E-tongue) analysis was performed according to the methods described by Chen et al. [46] and Peng et al. [47], with minor modifications. Briefly, 1.0 g of ZSS powder was extracted with 100 mL of distilled water in an ultrasonic bath, followed by centrifugation and filtration to obtain the test solution. The taste profile was analyzed using a TS-5000Z electronic tongue (Insent Inc., Kanagawa, Japan). The sensors were sequentially washed, equilibrated, and immersed in the sample solution to measure electric potential changes corresponding to basic tastes (sourness, bitterness, astringency, umami, and saltiness) and their aftertastes. The average values from the last three of four measurement cycles were used for data analysis.

### 4.8. GC-IMS Analysis Method

The volatile profile of ZSS was characterized using headspace gas chromatography-ion mobility spectrometry (GC-IMS) with a FlavourSpec^®^ instrument (G.A.S., Dortmund, Germany). Specifically, 1.0 g of powdered sample was accurately weighed into a 20 mL headspace vial, sealed, and equilibrated at 60 °C for 15 min. Subsequently, 500 μL of headspace vapor was automatically injected into the GC inlet in splitless mode at 45 °C. Chromatographic separation was achieved using an MXT-WAX capillary column (30 m × 0.53 mm ID, 1.0 μm df) with high-purity nitrogen as the carrier gas. The GC flow rate was programmed as follows: initial 2 mL/min held for 2 min, increased to 10 mL/min at 8 min, and finally raised to 100 mL/min until the end of the 15-min analysis. The column temperature was maintained at 60 °C throughout the analysis, while ionization was conducted at 45 °C. Compound identification was performed by matching both retention index (RI) and drift time against the built-in GC-IMS library. The RI of each volatile compound was calculated using an external calibration curve established with a C4–C9 n-ketone mixture (comprising 2-butanone, 2-pentanone, 2-hexanone, 2-heptanone, 2-octanone, and 2-nonanone) [48]. To further ensure the accuracy of the qualitative analysis, the calculated RIs were rigorously verified by comparing them with literature values from the NIST database.

### 4.9. Data Analysis

All experiments were conducted with three independent biological replicates. Statistical analysis was performed using Tukey’s test in SPSS software (version 22.0), with *p* < 0.05 considered statistically significant. Data are presented as mean ± standard deviation (SD). Graph generation was carried out using OriginPro (version 2021, OriginLab Corporation, Northampton, MA, USA).

## 5. Conclusions

This study systematically elucidates the comprehensive impact of harvest timing and stir-frying processing on the medicinal quality and flavor characteristics of ZSS. These findings confirm that harvesting at physiological maturity (15 September) is the prerequisite for maximizing the accumulation of bioactive secondary metabolites, including total flavonoids and saponins. Furthermore, the stir-frying process acts as a critical “efficacy-enhancing” step. While it induced a slight thermal degradation of saponins, stir-frying significantly enriched the content of spinosin and enhanced the antioxidant capacity. This specific chemical reconfiguration translated into superior sedative-hypnotic and lifespan-extending effects in the *Caenorhabditis elegans* model. In terms of sensory quality, thermal processing fundamentally reconstructed the flavor profile via the Maillard reaction, generating characteristic roasted aroma compounds such as 2-methylpyrazine. This effectively masked undesirable green notes and improved palatability by harmonizing bitterness with umami and richness. Collectively, this study provides a theoretical basis for the standardized production of high-quality ZSS, advocating for an integrative strategy that combines “optimal maturity harvesting” with “precise stir-frying” to simultaneously maximize therapeutic efficacy and sensory acceptance.

## Data Availability

The original contributions presented in this study are included in the article. Further inquiries can be directed to the corresponding authors.

## Supplementary Materials

The following supporting information can be downloaded at: https://www.mdpi.com/article/10.3390/plants15101573/s1, Table S1: Sensory Evaluation Form. Table S2: Determination of volatile flavor compounds of ZSS at before and after stir-fried points by GC-IMS. Figure S1: Representative HPLC chromatograms of reference standards and sample extracts. This figure includes the HPLC profiles for spinosin, jujuboside A, and jujuboside B with annotated retention times for both standards and sample extracts.
