# Supplementary material for: Impact of Harvest Timing and Stir-Frying on the Bioactive Compounds, Bioactivities, and Flavor of Ziziphi Spinosae Semen: An Integrated Analysis via GC-IMS, Electronic Sensors, and Caenorhabditis elegans Model
Source: Plants (Basel). 2026 May 21;15(10):1573. doi: 10.3390/plants15101573 (PMC13211273; doi:10.3390/plants15101573)

Figure S1. Representative HPLC chromatograms of reference standards and sample extracts. This figure includes the HPLC profiles for spinosin, jujuboside A, and jujuboside B with annotated retention times for both standards and sample extracts.

Standard

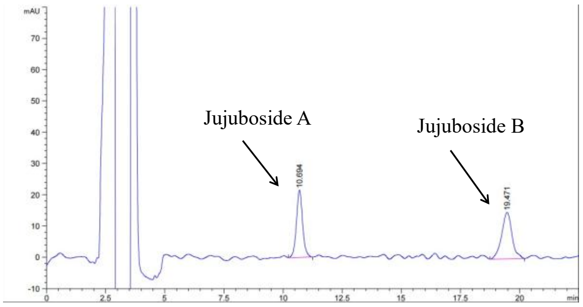

Sample

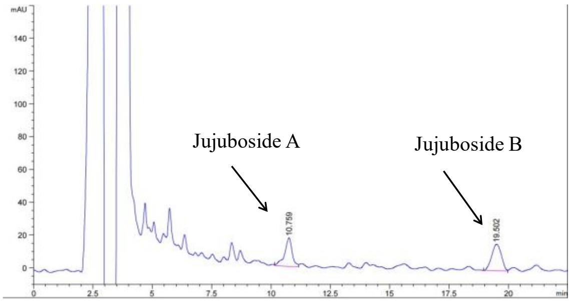

Standard

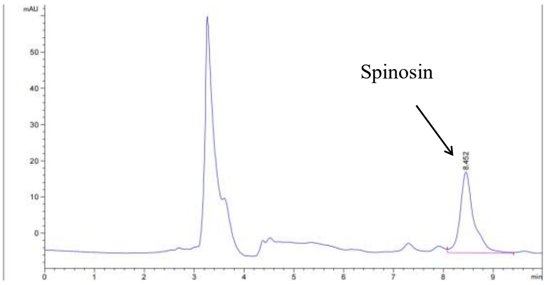

Sample

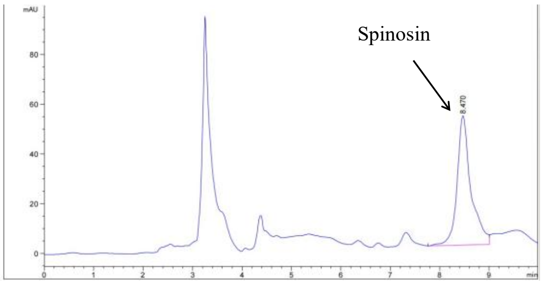

Supplement: Supplementary file 1 [file plants-15-01573-s001.zip › Figure S1.pdf]
